# Prevalence of antimicrobial-resistant *Escherichia coli* as an indicator bacterium in livestock and companion animals in Mongolia

**DOI:** 10.1016/j.onehlt.2026.101502

**Published:** 2026-06-27

**Authors:** Erdenebat Bulgan, Batsaikhan Chantsal, Tsognemekh Bolormaa, Badrakh Sandagdorj, Purevdorj Nyam-Osor, Batsukh Naranchimeg, Temuulen Erdenebat, Eisaku Kikuchi, Akio Suzuki, Jirachaya Toyting-Hiraishi, Toyotaka Sato, Motohiro Horiuchi

**Affiliations:** a Laboratory of Veterinary Hygiene, Graduate School of Infectious Diseases, Faculty of Veterinary Medicine, Hokkaido University, Japan; b Department of Veterinary Public Health, School of Veterinary Medicine, Mongolian University of Life Sciences, Mongolia; c Laboratory of Veterinary Sanitation and Hygiene, Ulaanbaatar Veterinary Department, Mongolia; d One Health Research Center, Hokkaido University, Japan; e Institute for Integrated Innovations, Hokkaido University, Japan; f Veterinary Research Unit, International Institute for Zoonosis Control, Hokkaido University, Japan; g Global Station for Zoonosis Control, Global Institute for Collaborative Research and Education, Hokkaido University, Japan

**Keywords:** Antimicrobial resistance, *Escherichia coli*, ST131, ST1193, Cefotaxime, Meropenem, Ciprofloxacin

## Abstract

In Mongolia, no national data are currently available on the status of antimicrobial-resistant (AMR) bacteria in livestock and companion animals. Given the need for international coordination in tackling antimicrobial resistance, clarifying the prevalence of AMR bacteria in animals is an urgent priority. In this study, we isolated *Escherichia coli*, as an indicator bacterium, from eight animal species, including livestock and companion animals, and examined their antimicrobial resistance to evaluate the current burden of AMR bacteria in animals in Mongolia. The prevalence of AMR *E. coli* was very low in livestock raised under nomadic systems (camels, goats, and sheep). AMR *E. coli* prevalence in cattle remained low, possibly due to a mix of nomadic and intensive husbandry practices. In contrast, AMR *E. coli* was highly prevalent in pigs and chickens raised by intensive husbandry. In chickens, 93.9% of *E. coli* isolates were AMR, and 67.4% exhibited multidrug resistance. In pigs, 70.3% of *E. coli* isolates were AMR, and 37.8% were multidrug-resistant. Rates of horses and dogs carrying AMR *E. coli* were also very high. In particular, animals in frequent contact with humans exhibited higher AMR *E. coli* carrying rates. Moreover, carbapenemase-producing *E. coli* and fluoroquinolone- and/or cephalosporin-resistant *E. coli* clonal groups ST131 and ST1193, which pose significant global public health threats, were identified only in owned dogs. To the best of our knowledge, this is the first report on the prevalence of AMR *E. coli* in livestock and companion animals in Mongolia. These findings highlight the urgent need for coordinated One Health interventions to reduce transmission risk between humans and companion animals.

## 1 Introduction

Antimicrobial resistance poses a threat to public health globally. Under the One Health concept, surveillance of antimicrobial resistant (AMR) organisms in humans, animals, foods, and environments, as well as antimicrobial use (AMU), is essential for identifying AMR risks and implementing effective measures to protect human and animal health, and preventing the transmission of AMR bacteria and/or horizontal transfer of resistance genes among animals, humans, and the environment [1], [2]. However, in resource-limited settings, particularly in low- and middle-income countries, insufficient capacity to address AMR challenges hinders the establishment of surveillance and reporting systems and limits AMR-related research [3], [4].

The tripartite AMR Country Self-Assessment Survey conducted by the World Health Organization (WHO), the Food and Agriculture Organization, and the World Organization for Animal Health reported insufficient capacity for AMR and AMU monitoring in veterinary medicine in Mongolia [5]. Currently, no official data are available on AMR prevalence and AMU in animals. Given the established correlation between AMU and AMR in food-producing animals [6], [7], an enhanced surveillance system for AMU and AMR bacteria is urgently required. Recent studies have described AMR cases in chicken and cattle infected with *Campylobacter* spp. and AMR contamination of poultry meat with *Salmonella Enteritidis* in Mongolia [8], [9], [10]. The high AMR rates in *Campylobacter* spp. in chickens; 100% and 50% of isolates were resistant to tetracycline and fluoroquinolones, respectively [9], underscore the urgent need to clarify the current AMR status in animals.

*E. coli*, a commensal bacterium, serves as a key indicator for AMR surveillance across human, animal, food, and environmental sectors because AMR levels in commensal *E. coli* closely reflect selection pressure induced by antibiotic use [11], [12], [13], [14]. Therefore, this study examined AMR in *E. coli* isolated from livestock and companion animals to assess the current burden of AMR bacteria in animal population in Mongolia.

## 2 Materials and methods

### 2.1 Sampling

Between March 2022 and April 2025, cloacal swabs were collected from chickens (*n* = 50) and rectal swabs were collected from camels (*n* = 19), goats (*n* = 69), sheep (*n* = 57), cattle (*n* = 100), horses (*n* = 30), pigs (*n* = 40), and dogs (*n* = 154) (Supplementary Table S1). Among the sampled horses, 20 were racing horses, and 10 were raised under nomadic husbandry. Among the sampled dogs, 124 were owned, and 30 were shelter dogs. While most swabs were collected from clinically healthy animals, samples were collected from 8 horses and 10 dogs showing clinical signs, including coughing, vomiting, and diarrhea. All sick horses tested were racing horses, and all sick dogs tested were owned dogs. Samples were collected in Ulaanbaatar City, and the Tuv, Gobisumber, and Dundgobi Provinces (Supplementary Fig. S1). Seven to 19 samples were collected at each farm.

### 2.2 Isolation of *E. coli*

Rectal and cloacal specimens were collected using sterile cotton swabs. Each swab was immediately placed into a tube containing 5 mL buffered peptone water (Oxoid, Basingstoke, UK) and transported to the laboratory within 5 h, except for camel samples, which took 1 day to reach the laboratory in Ulaanbaatar from the sampling point. One loop of 10-μL aliquots was inoculated onto antibiotic-free CHROMAgar ECC (CHROMAgar, Paris, France) and CHROMAgar ECC containing 1 μg/mL ciprofloxacin or 1 μg/mL cefotaxime (Tokyo Chemical Industry Co., Ltd., Tokyo, Japan) for the isolation of *E. coli*. Plates were incubated at 37 °C for 24 h. *E. coli* colonies were identified by their typical blue color on CHROMAgar ECC. Up to three colonies were selected from each agar plate and subcultured onto CHROMAgar ECC agar plates to obtain pure cultures. Suspected *E. coli* colonies underwent confirmation using Microflex matrix-assisted laser desorption ionization time-of-flight mass spectrometry (MALDI-TOF MS) using a MALDI Biotyper Compass (Bruker Daltonics, Bremen, Germany).

### 2.3 Antimicrobial susceptibility testing

Antimicrobial susceptibility was determined by broth microdilution method according to Clinical and Laboratory Standards Institute (CLSI) guidelines. Minimum inhibitory concentration (MIC) values were interpreted based on CLSI breakpoints (CLSI M100) [15]. The antibiotics used in this study were gentamicin (GEM), tetracycline (TET), chloramphenicol (CHL), ampicillin (AMP), cefazolin (CFZ), cefmetazole (CMZ), cefoxitin (CFX), cefotaxime (CTX), meropenem (MEM), nalidixic acid (NAL), ciprofloxacin (CIP), and trimethoprim/sulfamethoxazole (STX). *E. coli* ATCC 25922 was used for quality control in each experiment.

### 2.4 DNA extraction and genetic analyses

Bacterial genomic DNA was extracted using NucleoSpin Microbial DNA (Takara Bio, Shiga, Japan) according to the manufacturer's instructions. Major *β*-lactamase encoding genes were identified using multiplex PCR as described previously [16]. PCR was performed in a final volume of 25 μL, containing 12.5 μL of AmpliTaq Gold GC360 Master Mix (Thermo Fisher Scientific, Waltham, MA, USA), primers at specific concentrations in the previous paper [16], 1 μL of template DNA (approx. 80 ng), and Milli-Q (MQ) water up to 25 μL. Carbapenemase-encoding genes were identified by multiplex PCR for *bla*_IMP_, *bla*_NDM_, *bla*_KPC_, and *bla*_OXA-48_, as described previously [17]. Reaction mixture contained 12.5 μL of AmpliTaq Gold GC360 Master Mix (Thermo Fisher Scientific), 0.4 μM of each primer, 2 μL of template DNA (approx. 160 ng), and MQ water to achieve a final volume of 25 μL. *bla*_CTX-M_ group-specific genes (*bla*_CTX-M-1_, *bla*_CTX-M-2_, and *bla*_CTX-M-9_) were typed using PCR as described previously [16], [18]. PCR products were subjected to nucleotide sequencing for *bla*_CTX-M_ subtype identification. The obtained sequences were aligned using the online ClustalW program (https://www.genome.jp/tools-bin/clustalw) to analyze similarity with sequences available in the GenBank nucleotide database using the Beta-Lactamase Database (http://bldb.eu/). *E. coli* ST131 and ST1193 were determined using multiplex PCR as described elsewhere [19], [20]. Each reaction was prepared in a final volume of 20 μL containing 10 μL of Quick Taq (Toyobo, Osaka, Japan), 0.5 μM of each primer, 1 μL of template DNA (approx. 80 ng), and MQ water. O-genotype (Og) and H-genotype (Hg) were identified using multiplex PCR as described previously [21], [22]. The O25b genotype was determined using primers developed by Clermont et al [23]. Primers, amplicon sizes, and reaction conditions used were listed in Supplementary Table S2.

## 3 Results

### 3.1 Isolation of *E. coli*

Antibiotic-free CHROMAgar ECC agar plates (hereafter ECC) were used for the unbiased isolation of *E. coli*. Furthermore, CTX- or CIP-containing ECC were used to enhance the efficacy of isolating cephalosporin- or fluoroquinolone-resistant *E. coli*, respectively. If only one or two suspected colonies appeared on the agar plate, those colonies were isolated for pure culture. If more than three colonies grew, three colonies were randomly selected for pure culture; thus, a maximum of nine *E. coli* colonies per animal were collected from three agar plates. In total, 1110 *E. coli* colonies were isolated from 8 animal species (total: 398 animals) using antibiotic-free ECC (Supplementary Table S1) and used to estimate unbiased AMR rates. An additional 356 *E. coli* colonies were isolated from 77 and 53 animals, including 204 from CTX-containing ECC and 152 from CIP-containing ECC, respectively, which were included in some analyses. When explaining the results of analysis involving these isolates, we clearly stated that isolates used include the targeted isolates of resistant subpopulations. All isolates were confirmed to be *E. coli* by MALDI Biotyper.

### 3.2 Antimicrobial resistance of *E. coli* isolates

Unbiased AMR rates were calculated using the results of antimicrobial susceptibility testing of *E. coli* isolated using antibiotic-free ECC (*n* = 1110; Supplementary Table S1), as those were isolated under non-selection bias (Table 1). Isolates from camels and goats showed no resistance to any of the antibiotics tested, whereas a few sheep isolates were resistant to TET (2.5%) and AMP (1.8%). Cattle isolates demonstrated low resistance rates (0.5%–6.6%) to most antibiotics, including first-generation cephalosporins (CFZ), whereas no *E. coli* resistant to second-generation or higher cephalosporins (CMZ, CFX, and CTX) were isolated using antibiotic-free ECC. However, when CTX-containing ECC was used for isolation, 10 CTX-resistant *E. coli* were isolated from 4 cattle samples (Supplementary Tables S3 and S4), suggesting that some cattle carried CTX-resistant *E. coli* at low abundance. Higher AMR rates were observed in isolates from horses, pigs, chickens, and dogs. AMR rates among horse isolates were moderate or high for more than half of the antibiotics tested: rates of resistance to CTX, NAL, and CIP were moderate (10.9%–19.2%), whereas rates of resistance to TET, CHL, AMP, CFZ, and STX were high (21.9%–28.8%). Pig isolates showed high resistance to TET but moderate resistance to AMP, NAL, and STX (Table 1). However, when the targeted isolates of CTX- or CIP-resistant subpopulations obtained using CTX- and CIP-containing ECC (24 and 42 isolates, respectively; Supplementary Table S1) were combined with the 106 isolates from antibiotic-free ECC, the AMR rate of *E. coli* increased not only for CTX (6.6%–19.8%) and CIP (1.9%–28.4%) but also for other antibiotics, likely due to multidrug resistance (MDR; Table 1, Supplementary Table S3)*.* Chicken isolates showed extremely high resistance to AMP (85.6%), and with very high TET- and NAL-resistant rates at 55.6% and 58.9%, respectively. Rates of CHL-, CFZ-, CTX-, CIP-, and STX-resistant isolates were also high (33.3%–40.0%, unbiased AMR rates; Table 1). *E. coli* isolated from dogs also exhibited high AMR rates to multiple antibiotics: resistance to GEM and CIP was moderate (13.8% and 12.3%, respectively), while resistance to TET, AMP, CFZ, CTX, and STX was high (21.5%–40.4%, unbiased AMR rates; Table 1). *E. coli* resistant to second-generation cephalosporins (2nd-GC) CMZ and CFX, which was not observed in other animal species, were isolated from dogs at low levels (2.3% and 5.4%, respectively). Notably, *E. coli* resistant to MEM, an antibiotic not used in veterinary medicine in Mongolia, was detected only in dog isolates, even using antibiotic-free ECC (1.9%, unbiased AMR rates; Table 1).

**Table 1:** Antimicrobial resistant rates of *E. coli* isolates (unbiased prevalence estimates).

Antibiotics^1^	Species								Total (n = 1110)
	Camel (*n* = 22)^2^	Goat (*n* = 185)	Sheep (*n* = 163)	Cattle (*n* = 211)	Horse (*n* = 73)	Pig (*n* = 106)	Chicken (*n* = 90)	Dog (*n* = 260)	
GEM	0	0	0	2 (0.9)^3^	5 (6.8)	3 (2.8)	3 (3.3)	36 (13.8)	49 (4.4)
TET	0	0	4 (2.5)	14 (6.6)	21 (28.8)	42 (39.6)	50 (55.6)	84 (32.3)	215 (19.4)
CHL	0	0	0	3 (1.4)	18 (24.7)	1 (0.9)	30 (33.3)	20 (7.7)	72 (6.5)
AMP	0	0	3 (1.8)	5 (2.4)	20 (27.4)	17 (16.0)	77 (85.6)	105 (40.4)	227 (20.5)
CFZ	0	0	0	1 (0.5)	16 (21.9)	5 (4.7)	32 (35.6)	58 (22.3)	112 (10.1)
CMZ	0	0	0	0	0	0	0	6 (2.3)	6 (0.5)
CFX	0	0	0	0	0	0	0	14 (5.4)	14 (1.3)
CTX	0	0	0	0	14 (19.2)	7 (6.6)	35 (38.9)	56 (21.5)	112 (10.1)
MEM	0	0	0	0	0	0	0	5 (1.9)	5 (0.5)
NAL	0	0	0	9 (4.3)	9 (12.3)	18 (17.0)	53 (58.9)	61 (23.5)	150 (13.5)
CIP	0	0	0	3 (2.4)	8 (10.9)	2 (1.9)	36 (40.0)	32 (12.3)	94 (8.5)
STX	0	0	0	3 (1.4)	18 (24.7)	13 (12.3)	35 (38.9)	71 (27.3)	140 (12.6)

1 GEM, gentamicin; TET, tetracycline; CHL, chloramphenicol; AMP, ampicillin; CFZ, cefazolin; CMZ, cefmetazole; CFX, cefoxitin; CTX, cefotaxime; MEM, meropenem; NAL, nalidixic acid; CIP, ciprofloxacin; SXT, sulfamethoxazole/trimethoprim.

2 Number of *E. coli* isolated from each animal species using antibiotic-free ECC (n = 1,110; Supplementary Table 1).

3 Numbers in parentheses indicate percentages of the AMR *E. coli* isolates to the total *E. coli* isolates from each species using antibiotic-free ECC.

**Fig. 1:**
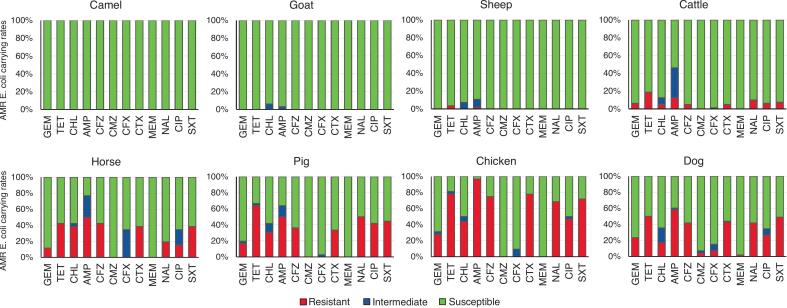
Antimicrobial-resistant *E. coli* carrying rates in each animal species. Animals from which at least one *E. coli* was isolated using antibiotic-free and/or antibiotic-containing CHROMAgar ECC plates were used for calculation (total: 1466 isolates including targeted isolates of CTX- or CIP-resistant subpopulations from 403 animals; Supplementary Table S1). Even if more than two different types of antimicrobial-resistant *E. coli* were isolated from one animal, e.g., different multidrug resistance patterns, the animal was counted as one antimicrobial-resistant *E. coli*-positive animal for each antibiotic. Resistant, intermediate, and susceptible isolates to each antibiotic were determined using the CLSI guidelines based on the minimum inhibitory concentrations determined using the microdilution assay. GEM, gentamicin; TET, tetracycline; CHL, chloramphenicol; AMP, ampicillin; CFZ, cefazolin; CMZ, cefmetazole; CFX, cefoxitin; CTX, cefotaxime; MEM, meropenem; NAL, nalidixic acid; CIP, ciprofloxacin; SXT, sulfamethoxazole/trimethoprim.

**Fig. 2:**
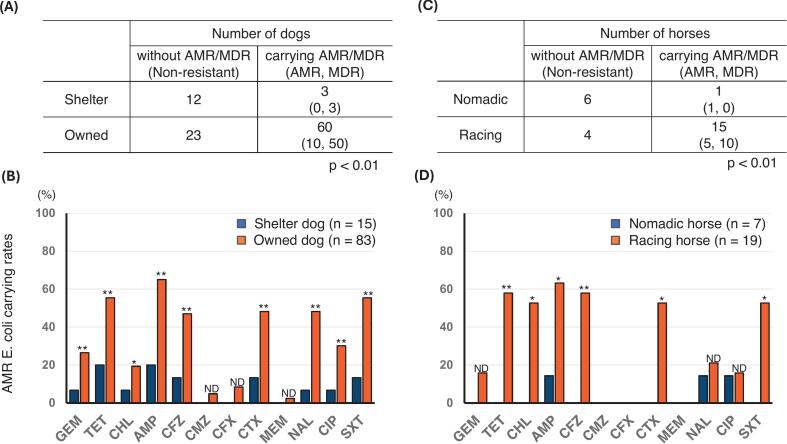
Comparison of AMR *E. coli* carrying rates in animals with frequent and infrequent human contact. (A) Number of owned and shelter dogs, and (B) racing and nomadic horses carrying AMR/MDR or without AMR/MDR. Numbers in the parentheses indicate animals carrying AMR and MDR. Isolates resistant to ≥3 antimicrobial classes were defined as MDR *E. coli*. Isolates resistant to ≤2 antimicrobial classes were classified as AMR. Isolates that are neither MDR nor AMR were classified as non-resistant, including those defined as susceptible and intermediate. Eight antibiotic classes were used for determining AMR and MDR (Supplementary Table S5). Fishers exact test was used for statistical analysis. (C) AMR carrying rates in owned and shelter dogs and (D) in nomadic and racing horses. **: *p* < 0.01; *: *p* < 0.05 by Fishers exact test. ND: statistical analysis was not performed due to low numbers of AMR *E. coli* (sum of the AMR *E. coli* isolates was less than 10)*.* GEM, gentamicin; TET, tetracycline; CHL, chloramphenicol; AMP, ampicillin; CFZ, cefazolin; CMZ, cefmetazole; CFX, cefoxitin; CTX, cefotaxime; MEM, meropenem; NAL, nalidixic acid; CIP, ciprofloxacin; SXT, sulfamethoxazole/trimethoprim.

Fig. 1 shows rates of AMR *E. coli* carrying animals. To maximize the estimation of carriage rates, 1466 isolates, including those from CXT- and CIP-containing ECC as targeted isolates of CTX- or CIP-resistant subpopulations, were used based on data in Supplementary Table S4, incorporating isolates categorized as “intermediate” based on MIC values. Neither camels nor goats carried *E. coli* resistant to any of the antibiotics tested. Only a small percentage of sheep carried TET- or AMP-resistant *E. coli.* Cattle harbored AMR *E. coli* against several antibiotics, although the AMR carrying rates were generally low except for AMP and TET. In contrast, substantially higher carriage rates of AMR *E. coli* were observed in horses, pigs, chickens, and dogs. Notably, 4, 7, and 2 dogs carried CMZ- (4.1%), CFX- (7.1%), and MEM-resistant *E. coli*, respectively (Supplementary Table S4), all of which were owned dogs (Fig. 2). When comparing between owned and shelter dogs, presumed to have frequent and infrequent human contact, respectively, number of owned dogs carrying AMR/MDR was higher than shelter dogs (Fig. 2A, *p* < 0.01, Fisher's exact test). As expected, owned dogs exhibited higher AMR carrying rates than shelter dogs for variety of antibiotics (Fig. 2B). A similar tendency was observed in racing horses which have more frequent contact with humans than nomadic horses; number of racing horses carrying AMR/MDR was higher than nomadic horses (Fig. 2C, *p* <0.01, Fisher's exact test) and racing horses exhibited higher AMR carrying rates than nomadic horses (Fig. 2D).

### 3.3 MDR of *E. coli* isolates

**Fig. 3:**
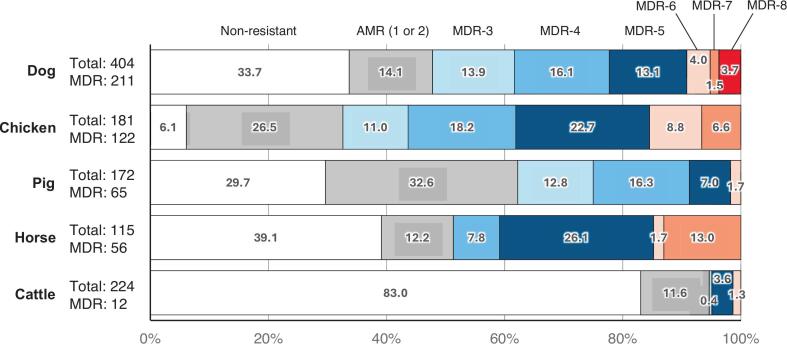
Presence of multidrug-resistant *E. coli.* Total *E. coli* isolates using antibiotic-free and antibiotic-containing CHROMAgar ECC plates were used for assignment (total 1466 isolates including targeted isolates of CTX- or CIP-resistant subpopulation; Supplementary Table S1). Isolates resistant to ≥3 antimicrobial classes were defined as MDR *E. coli*. Isolates resistant to ≤2 antimicrobial classes were classified as AMR. Isolates that are neither MDR nor AMR were classified as non-resistant, including those defined as susceptible and intermediate.

MDR of the isolates was evaluated using 8 antibiotic classes, including Aminoglycosides (AG), Tetracyclines (TET), Amphenicols (CMP), Penicillins (PEN), Cephalosporins (CEP), Carbapenems (CP), Fluoroquinolones (FQ), and Sulfonamides (SUL), according to the previous report [24]. To identify the maximum MDR patterns, 1466 isolates, including those from CXT- and CIP-containing ECC as targeted isolates of CTX- or CIP-resistant subpopulations, were used in this analysis. Forty-eight distinct MDR patterns were identified (Supplementary Table S5). More than half of the dog isolates were MDR, and 15 isolates from 2 dogs were resistant to all 8 antibiotic classes (MDR-8), including CP (Fig. 3, Supplementary Table S5). Chicken isolates showed the highest MDR rates among the animal species tested (67.4%). *E. coli* resistant to 7 antibiotic classes (MDR-7) was found in horses, dogs, and chickens (Fig. 3). MDR rates were high in horse (48.7%) and pig (37.8%) isolates but low in cattle isolates.

### 3.4 Identification of β-lactamase genes

CTX-M-type extended-spectrum *β*-lactamases (ESBL) are the dominant mechanism of third-GC resistance [25]. Their spread is largely plasmid-mediated, and analyzing their subtypes helps elucidate the transmission routes of *ESBL* genes and ESBL-producing *E. coli* among animals in Mongolia. One to four isolates were chosen from individual animals, depending on their AMR profiles, and in total, 151 CTX-resistant *E. coli* isolates were analyzed. Six *β*-lactamase genes were identified among 18 detectable by multiplex PCR (Table 2). As expected, the most common *β*-lactamase gene was *bla*_CTX-M_. *bla*_TEM_ was detected in horse, cattle, dog, and chicken isolates but not in pig isolates. *bla*_SHV_ was detected in pig, chicken, and dog isolates. Furthermore, *bla*_OXA_ was identified in 1 chicken isolate, and *bla*_DHA_ was detected in 2 dog isolates. All MEM-resistant isolates carried *bla*_NDM-1_ (Table 2).

**Table 2:** Identification of *β*-lactamase genes in cephalosporin- and/or carbapenem-resistant *E. coli* isolates.

Species	Isolate number^1^	*β*-lactamase genes					
		*bla*_TEM_	*bla*_SHV_	*bla*_OXA_	*bla*_CTX-M_	*bla*_DHA_	*bla*_NDM-1_
Cattle	5	3 (60.0)^2^	0	0	5 (100)	0	0
Horse	19	12 (63.2)	0	0	19 (100)	0	0
Pig	22	0	9 (40.9)	0	10 (45.5)	0	0
Chicken	42	9 (21.4)	7 (16.6)	1 (2.4)	30 (71.4)	0	0
Dog	63	29 (46.0)	2 (3.2)	0	59 (93.7)	2 (3.2)	6 (9.5)

1 Number of cephalosporin- and/or carbapenem-resistant *E. coli* isolates used for analysis

2 Numbers indicate number of isolates positive for each gene and numbers in parentheses indicate percentages of isolates carrying each corresponding gene.

Nucleotide sequence analysis classified *bla*_CTX-M_ genes into the *bla*_CTX-M-1_- and *bla*_CTX-M-9_-groups (Supplementary Table S6). More than half of the isolates carried *bla*_CTX-M-1_-group genes (80/123), with *bla*_CTX-M-55_ as the most frequently detected subtype (45/80). Dog isolates carried various *bla*_CTX-M_ subtypes*.* Most chicken isolates carried the *bla*_CTX-M-55_ subtype. *bla*_CTX-M-1_ was detected only in horse isolates, whereas *bla*_CTX-M-3_ was found only in dog isolates. Three subtypes were identified in *bla*_CTX-M-9_-group: *bla*_CTX-M-14_, *bla*_CTX-M-27_, and *bla*_CTX-M-65_.

### 3.5 Identification of *E. coli* sequence types (ST)131 and ST1193 in dogs isolates

Isolates of extraintestinal pathogenic *E. coli* (ExPEC) clonal groups ST131 and ST1193 are often resistant to critically important antimicrobials, such as FQ and CEP. Since FQ- and/or CEP-resistant *E. coli* ST131 and ST1193 cause urinary tract and bloodstream infection in humans, they pose a global public health concern [26], [27]. Inter-species transmission, particularly between humans and companion animals, has been implicated in the maintenance of FQ-resistant ST131 and ST1193 [26], [28], [29]. Thus, we analyzed 135 CIP- and/or CTX-resistant *E. coli* isolated from cattle (*n* = 5), horses (*n* = 19), chickens (*n* = 28), pigs (*n* = 23), and dogs (*n* = 60) for ST131 and ST1193 using multiplex PCR. Four and 2 isolates from owned dogs were identified as ST131 and ST1193, respectively (Table 3). All 6 isolates were MDR *E. coli* resistant to ≥4 antibiotic classes, including FQ and/or CEP. One ST131 isolate (ID: db4–1ctx), which was isolated on CTX-containing ECC, was susceptible to FQ and exhibited Og- and Hg-types (O16:H5) that differed from those of other ST131 isolates (O25b:H4; Table 3). Neither ST131 nor ST1193 was identified in other animals than dogs.

**Table 3:** Identification of ST131 and ST1193 among fluoroquinolone- and/or cephalosporin-resistant *E. coli*.

Species	Isolate ID	Health status	O:H genotypes	ST^1^	Clade	Subclade	Susceptibility to antibiotics classes^4^								*β*-lactamase genes
							AM	TET	CMP	FQ	PEN	CEP	CP	SUL	
Dog (owned)	db4–1 ctx	Healthy	O16:H5	ST131	N·D^2^	N·D	S^5^	R	S	S	R	R	S	R	*bla*_CTX-M-14_, *bla*_TEM_
Dog (owned)	AD-7 ctx-2	Healthy	O25b:H4	ST131	C	C1-M27	S	R	S	R	R	R	S	R	*bla*_CTX-M-27_, *bla*_TEM_
Dog (owned)	AD-8 ecc-2	Healthy	O25b:H4	ST131	C	C1-M27	S	R	S	R	R	R	S	R	*bla*_CTX-M-27_
Dog (owned)	d54–1 ctx	Healthy	O25b:H4	ST131	C	C2	R	S	S	R	R	R	S	S	*bla*_CTX-M-15_, *bla*_SHV_
Dog (owned)	D28–1 ecc	Healthy	O75:H5	ST1193	N.T^3^	N.T	R	S	S	R	R	R	S	R	*bla*_CTX-M-64_, *bla*_TEM_
Dog (owned)	D1–2 ecc	Sick	O75:H5	ST1193	N.T	N.T	R	R	S	R	R	R	S	R	*bla*_CTX-M-64_, *bla*_SHV_

1 Sequence type.

2 Not determined.

3 Not tested.

4 AG: Aminoglycosides, TET: Tetracyclines, CMP: Amphenicols, FQ: Fluoroquinolones, PEN: Penicillins, CEP: Cephalosporins, CP: Carbapenems, SUL: Sulfonamides.

5 S, susceptible; R, resistant.

## 4 Discussion

Our results clearly demonstrate that the prevalence of AMR *E. coli* was very low in livestock raised under nomadic systems (camels, goats, and sheep). In contrast, AMR *E. coli* were extremely highly prevalent in livestock raised under intensive husbandry (pigs and chickens). AMR prevalence in cattle remained low, possibly due to a mix of nomadic and intensive husbandry practices. This pattern mirrors our previous findings for *Campylobacter* spp., in which AMR rates were very high in chicken isolates but very low in cattle isolates [8], [9]. Currently, Mongolia lacks a fully implemented veterinary prescription system but various classes of antibiotics, such as PEN, SUL, TET, AG, FQ, CEP, Macrolides, and Polymyxins can be purchased over the counter at veterinary pharmacies. Moreover, official antimicrobial use/sales data are unavailable. Consequently, linking AMU to the AMR pattern observed in animals is difficult. However, CIP and enrofloxacin (FQ), CFZ (1st-GC), and ceftiofur and CTX (3rd-GC) are used in veterinary medicine (personal communication from veterinary practitioners who helped fecal sampling from dogs and horses). The use of those antibiotics in veterinary clinical settings might have some impact on the high resistance rates to CFZ and CTX but low resistance rates or no resistance to 2nd-GC, such as CMZ and CFX, in *E. coli* isolated from dogs and horses (Table 1). We have not obtained any evidence on the use of FQ and CEP in chicken farms so far; however, since AMU in animals is one of the factors contributing to the emergence of AMR bacteria [30], the high levels of FQ- and CEP-resistant *E. coli* in chickens, dogs, and horses (Table 1) clearly demonstrate the urgent need to strengthen measures against AMR in veterinary settings. This study further revealed that dogs and horses with frequent human contact exhibited higher rates for AMR *E. coli* isolation (Fig. 2). Similar trend has been reported in the surveillance of AMR *E. coli* in shelter dogs in Japan [31].

In this study, *E. coli* isolated from chicken showed high AMR rates to various antibiotics, including critically important antibiotics for human medicine such as CTX and CIP (38.9% and 40.0%, respectively; Table 1). By contrast, AMR rates to CTX and CIP in pig isolates were moderate (6.6% and 1.9%, respectively; Table 1) and those in cattle isolates were low (0% and 2.4%, respectively; Table 1). The pattern of higher 3rd-GC and FQ resistance in chicken compared with pig isolates, and less frequent resistance to those antibiotics in cattle isolates, has been reported in China, Korea, and Japan [32], [33], [34], [35]. The CTX/3rd-GC resistance rate in chicken isolates (38.9%) was comparable to that reported for China (21.3%–46.7%) [34], [35] but higher than rates reported for Korea (8.8%) [33] and Japan (3.9%) [32]. The FQ resistance rate in chicken isolates (40.0%) exceeded that reported for Japan (16.1%) [32] but was lower than reported rates in China (55.6%–68.8%) [34], [35] and Korea (76.1%) [33]. For pig isolates, the CTX/3GC resistance rate in Mongolia (6.6%) was lower than that reported for China (29.6%) [36] and Korea (12.7%) [33] but higher than Japan (0.0%) [32]. FQ resistance rate (1.9%) was lower than reported rates in China (51.1%–55.6%) [34], [35] and Korea (76.1%) [33] but higher than Japan (0.7%) [32]. Compared with EU medians, the CTX resistance rate in Mongolian pig isolates (6.6%) was exceeded the EU median (0.8%), whereas the CIP resistance rate (1.9%) was below the EU median (7.7%) [37]. Differences in the prevalence of AMR *E. coli* across countries/regions and livestock species likely reflect a complex interplay of factors, including variations in AMU, regulatory frameworks, and husbandry practices in each country and region.

AMR rates to CTX and CIP in dog isolates in Mongolia (21.5% and 12.3%, respectively) were comparable to or slightly higher rates reported for healthy dog in some Asian countries, such as Japan (CXT and CIP resistance rate: 11.4% and 12.9% [38]) and Korea (3rd-GC and enrofloxacin resistance rate: 6.0%–16.5% and 16.2% [39]), but lower than rates reported in China [40]. However, presence of MEM-resistant *E. coli* and FQ- and/or CEP-resistant ST131 and ST1193 in owned dogs (Fig, 1 and Table 3), together with the higher rate of AMR *E. coli* carriage in dogs with frequent human contact (Fig, 2), highlights the need to raise public awareness and promote measures that reduce transmission risk between humans and companion animals.

Nucleotide sequence analysis classified *bla*_CTX-M_ genes revealed that more than half of the isolates carried *bla*_CTX-M-1_-group genes (80/123), with *bla*_CTX-M-55_ as the most frequently detected subtype (45/80) (Supplementary Table S6). *bla*_CTX-M-55_ was first reported in Thailand and is now the most predominant *bla*_CTX-M_ subtype in *E. coli* in livestock across Aisa, particularly in China [41]. Although further genetic analyses are required, the dominance of *bla*_CTX-M-55_ in chicken isolates in Mongolia suggests regional dissemination of this subtype through Asian countries. Dog isolates carried various *bla*_CTX-M_ subtypes, including *bla*_CTX-M-64_, which is a hybrid of the *bla*_CTX-M-1_ and *bla*_CTX-M-9_ groups [42].

It has been reported that *E. coli* isolates from patients with urinary tract infection (uropathogenic *E. coli*, UPEC) or diarrheal diseases (Diarrheagenic *E. coli*, DEC) in Mongolia exhibited an extremely high resistance rate to 1st-GC (∼85%), and high resistance rates to 3rd-GC (∼40%) and FQ (∼40%) [43], [44]. Moreover, more than 60% of DEC isolates were MDR *E. coli* [44]. FQ- and CEP-resistant *E. coli* human clinical isolates were first isolated in Mongolia in 2013 [45], indicating long-standing circulation of MDR *E. coli* against critically important antimicrobials in the community. Given that dogs and horses with frequent human contact carry AMR/MDR *E. coli* against critically important antimicrobials for human medicine, detailed comparative genetic analyses using whole-genome sequencing are required to elucidate the relationship between human and animal isolates.

Carbapenemase-producing *Enterobacterales* (CRE) pose significant global public health threats due to limited therapeutic options and high mortality [46] and are classified as a critical group of the WHO bacterial priority pathogen list [47]. Incidence of CRE in companion animals, food-producing animals, and retail meat are increasing worldwide [48], [49]. Furthermore, carbapenemase genes may also be transferred from humans to livestock [50]. In the present study, no MEM-resistant *E. coli* was isolated from any of the livestock examined, suggesting that the MEM-resistant *E. coli* isolates from owned dogs would not originate from food-producing animals in Mongolia. On the other hand, several reports have pointed out the possible transmission of carbapenemase-producing *E. coli* between humans and dogs in household and veterinary clinical settings [51], [52]. The fact that imipenem-resistant UPEC has been identified in Mongolia [43] and the use of Carbapenems in human medicine [53] suggest the importance of further monitoring and genetic analysis of Carbapenems-resistant *E. coli* isolates in humans and dogs.

ExPEC ST131 and ST1193 clonal groups, which exhibit FQ- and/or CEP-resistance, have also become a global public health concern because their MDR limits treatment options [26], [27]. In this study, three FQ- and CEP-resistant ST131 clade C with O25b:H4 carrying *bla*_CTX-M15_ or *bla*_CTX-M27_ were detected exclusively in owned dogs (Table 3). Since the late 2000s, ST131 clade B (ST131-B) carrying *bla*_CTX-M-15_ with O25b:H4 serotype has been predominantly isolated from humans in Europe and North America [54], [55], [56] and from pet dogs [57], [58]. ST131-B variants acquired FQ resistance were classified as ST131 clade C, which subsequently diversified [27]. The ST131 C1-M27 lineage carrying *bla*_CTX-M-27_ has also been isolated from humans and companion animals [59], [60], [61], [62]. In Mongolia, ST131 with O25b:H4 carrying *bla*_CTX-M-15_ or *bla*_CTX-M-27_ has already been detected in human clinical specimens [45]. Moreover, FQ- and CEP-resistant *E. coli* ST1193, which has been rapidly emerged in the global human population [27], [63] and reports of its isolation from companion animals have recently increased [29], [64], [65], was also identified in owned dogs (Table 3). As with ST131, FQ- and CEP-resistant ST1193 has also been detected in human clinical specimens in Mongolia [45]. These indicate the requirement of phylogenetic analyses using whole-genome-sequencing to elucidate possible transmission of ST131 and ST1193 between humans and companion animals.

This study has several limitations. The sampling area was restricted to the vicinity of Ulaanbaatar; therefore, the results may not reflect nationwide trends. Detailed genetic analysis using whole-genome sequencing remains imperative for future studies. Nevertheless, to the best of our knowledge, this is the first report on the prevalence of AMR *E. coli* in livestock and companion animals in Mongolia, highlighting the transmission risk of AMR bacteria to humans through the food chain and through frequent interaction with companion animals. In particular, the presence of carbapenemase-producing *E. coli* and *E. coli* clonal groups ST131 and ST1193 in dogs underscores the urgent need for coordinated One Health measures between human and veterinary medicine to reduce the transmission risk between humans and companion animals.

## 5 Conclusion

This study found that the prevalence of AMR *E. coli* is low among Mongolian livestock, specifically camels, sheep, and goats, which are primarily raised in a nomadic husbandry. In contrast, AMR *E. coli* prevalence is extremely high among chickens and pigs raised under intensive husbandry systems. The proportions of horses and dogs carrying AMR *E. coli* were also very high, particularly in animals known to have frequent human contact. This study also confirmed the presence of carbapenemase-producing *E. coli* and FQ- and/or CEP-resistant *E. coli* clonal groups ST131 and ST1193 in pet dogs. Furthermore, this study characterized current AMR *E. coli* trends across animal species in Mongolia and identified immediate targets for implementing countermeasures to reduce the risk of direct or indirect transmission of AMR bacteria between humans and animals.

## CRediT authorship contribution statement

**Erdenebat Bulgan:** Writing – original draft, Methodology, Investigation. **Batsaikhan Chantsal:** Resources, Investigation. **Tsognemekh Bolormaa:** Resources, Investigation. **Badrakh Sandagdorj:** Resources, Investigation. **Purevdorj Nyam-Osor:** Resources, Investigation, Conceptualization. **Batsukh Naranchimeg:** Resources, Investigation. **Temuulen Erdenebat:** Resources, Investigation. **Eisaku Kikuchi:** Investigation. **Akio Suzuki:** Methodology, Investigation. **Jirachaya Toyting-Hiraishi:** Writing – review & editing. **Toyotaka Sato:** Writing – review & editing, Methodology, Investigation. **Motohiro Horiuchi:** Writing – review & editing, Supervision, Project administration, Funding acquisition, Conceptualization.

## Ethics statement

The research using animal samples was approved by the Ethical Committee of Veterinary Science and Bio-Medical Research of the Mongolian University of Life Sciences (approval no. 22/01–02).

## Declaration of generative AI and AI-assisted technologies in the writing process

The authors did not use any generative AI during the preparation of this manuscript.

## Funding

This work was funded by International Collaborative Research (JSPS KAKENHI Grant Number 24KK0134) and the World‑leading Innovative and Smart Education (WISE) Program (1801) from the Ministry of Education, Culture, Sports, Science, and Technology, Japan.

## Declaration of competing interest

The authors declare that they have no competing interests.

## Data Availability

All data generated or analyzed during this study are included in this published article
