# Supplementary material for: Prevalence of antimicrobial-resistant Escherichia coli as an indicator bacterium in livestock and companion animals in Mongolia
Source: One Health. 2026 Jun 27;23:101502. doi: 10.1016/j.onehlt.2026.101502 (PMC13330623; doi:10.1016/j.onehlt.2026.101502)
Supplement: Supplementary file 1 — Supplementary material 1 [file mmc1.docx]

| Species | Farm No. | Animal No. used for isolation | Animal No.  *E. coli* isolated | Isolate number  (number of animal) | | | |
| --- | --- | --- | --- | --- | --- | --- | --- |
|  |  |  |  | ECC^1)^ | ECC-CTX^2)^ | ECC-CIP^3)^ | Total |
| Camel | 1 | 19 | 11 | 22  (11) | 0  (0) | 0  (0) | 22 |
| Goat | 6 | 69 | 65 | 185  (65) | 0  (0) | 0  (0) | 185 |
| Sheep | 6 | 57 | 55 | 163  (55) | 0  (0) | 0  (0) | 163 |
| Cattle | 10 | 100 | 80 | 211  (80) | 10  (4) | 3  (1) | 224 |
| Horse | 3^4)^ | 30 | 26 | 73  (25) | 30  (10) | 12  (4) | 115 |
| Pig | 4 | 40 | 36 | 106  (36) | 24  (9) | 42  (15) | 172 |
| Chicken | 5 | 50 | 32 | 90  (31) | 54  (19) | 37  (13) | 181 |
| Dog | 4^5)^ | 154 | 98 | 260  (95) | 86  (35) | 58  (20) | 404 |
| Total | 39 | 519 | 403 | 1,110  (398) | 204  (77) | 152  (53) | 1,466 |

Supplementary Table S1. Isolation of *E. coli* from various animal species in Mongolia.

^1)^ Isolates using CHROMAgar ECC agar plate without antibiotics that were used for calculating unbiased AMR rates.

^2)^ Isolates using CHROMAgar ECC agar plate supplemented with 1μg/mL cefotaxime (CTX) that were used as targeted isolates of CTX-resistant subpopulations.

^3)^ Isolates using CHROMAgar ECC agar plate supplemented with 1μg/mL ciprofloxacin (CIP) that were used as targeted isolates of CIP-resistant subpopulations.

^4)^ Number of clinics and farms where rectal swabs were collected

^5)^ Total number of clinics and a dog shelter where rectal swabs were collected

**Supplementary Table 2. Primer sequences and expected amplicon sizes for each PCR primer set used in this study.**

| Multiplex/simplex PCR for major β-lactamase genes (30 cycles of 94°C 40 sec, 60°C 40 sec, 72°C for 1 min):   - 55°C for amplification of *bla*_VIM_, *bla*_IMP_ and *bla*_KPC_ genes - 57°C for amplification of *bla*_GES_ and *bla*_OXA-48_ genes | | | | |
| --- | --- | --- | --- | --- |
| Target gene | **Primer name** | **Sequence (5′–3′)** | **Amplicon size (bp)** | **Reference** |
| TEM variants | MultiTSO-T_for | CATTTCCTGTCGGCCTTATTC | 800 | Dallenne et al. 2010 |
|  | MultiTSO-T_rev | CGTTCATCCATAGTTGCCTGA |  |  |
| SHV variants | MultiTSO-S_for | AGCCGCTTGAGCAAATTAAAC | 713 | Dallenne et al. 2010 |
|  | MultiTSO-S_rev | ATCCCGCAGATAAATCACCAC |  |  |
| OXA-1-like | MultiTSO-O_for | GGCACCAGATTCAACTTTCAAG | 640 | Dallenne et al. 2010 |
|  | MultiTSO-O_rev | GACCCCAAGTTTCCTGTAAGTG |  |  |
| CTX-M Group 1 | MultiCTXM Gp1_for | TTAGGAARTGTGCCGCTGYA**^1)^** | 688 | Dallenne et al. 2010 |
|  | MultiCTXM Gp1-2_rev | CGATATCGTTGGTGGTTRCCAT |  |  |
| CTX-M Group 2 | MultiCTXM Gp2_for | CGTTAACGGCACGATGAC | 404 | Dallenne et al. 2010 |
|  | MultiCTXM Gp1-2_rev | CGATATCGTTGGTGGTTRCCAT |  |  |
| CTX-M Group 9 | MultiCTXM Gp9_for | TCAAGCCTGCCGATCTGGT | 561 | Dallenne et al. 2010 |
|  | MultiCTXM Gp9_rev | TGATTCTCGCCGCTGAAG |  |  |
| CTX-M Group 8/25 | CTX-M Gp8/25-F | AACRCRCAGACGCTCTAC | 326 | Dallenne et al. 2010 |
|  | CTX-M Gp8/25-R | TCGAGCCGGAASGTGTYAT |  |  |
| DHA-1 and DHA-2 | MultiCaseDHA_for | TGATGGCACAGCAGGATATTC | 997 | 16 |
|  | MultiCaseDHA_rev | GCTTTGACTCTTTCGGTATTCG |  |  |
| CMY-2 group (CIT) | MultiCaseCIT_for | CGAAGAGGCAATGACCAGAC | 538 | 16 |
|  | MultiCaseCIT_rev | ACGGACAGGGTTAGGATAGY |  |  |
| EBC (AmpC) | MultiCaseEBC_for | CGGTAAAGCCGATGTTGCG | 683 | 16 |
|  | MultiCaseEBC_rev | AGCCTAACCCCTGATACA |  |  |
| GES-1 to GES-9, GES-11 | MultiGES_for | AGTCGGCTAGACCGGAAAG | 399 | 16 |
|  | MultiGES_rev | TTTGTCCGTGCTCAGGAT |  |  |
| PER-1, PER-3 | MultiPER_for | GCTCCGATAATGAAAGCGT | 520 | 16 |
|  | MultiPER_rev | TTCGGCTTGACTCGGCTGA |  |  |
| VEB-1 to VEB-6 | MultiVEB_for | CATTTCCCGATGCAAAGCGT | 648 | 16 |
|  | MultiVEB_rev | CGAAGTTTCTTTGGACTCTG |  |  |
| GES-1 to GES-9, GES-11 | MultiGES_for | AGTCGGCTAGACCGGAAAG | 399 | 16 |
|  | MultiGES_rev | TTTGTCCGTGCTCAGGAT |  |  |
| OXA-48-like | MultiOXA-48_for | GCTTGATCGCCCTCGATT | 281 | 16 |
|  | MultiOXA-48_rev | GATTTGCTCCGTGGCCGAAA |  |  |
| IMP variants | MultiIMP_for | TTGACACTCCATTTACDG | 139 | 16 |
|  | MultiIMP_rev | GATYGAGAATTAAGCCACYCT |  |  |
| VIM variants (including VIM-1, VIM-2) | MultiVIM_for | GATGGTGTTTGGTCGCATA | 390 | 16 |
|  | MultiVIM_rev | CGAATGCGCAGCACCAG |  |  |
| KPC-1 to KPC-5 | MultiKPC_for | CATTCAAGGGCTTTCTTGCTGC | 538 | 16 |
|  | MultiKPC_rev | ACGACGGCATAGTCATTTGC |  |  |

| *bla*_CTX-M_ PCR for group 1/2/9 typing - DNA sequencing (30 cycles of 95°C 30 sec, 60°C 30sec, and 72°C 1min) | | | | |
| --- | --- | --- | --- | --- |
| Target gene | **Primer name** | **Sequence (5′–3′)** | **Amplicon size (bp)** | **Reference** |
| CTX-M Group 1 | M13U | GGTTAAAAAATCACTGCGTC | 864 | 18 |
|  | M13L | TTGGTGACGATTTTAGCCGC |  |  |
| CTX-M Group 2 | M25U | ATGATGACTCAGAGCATTCG | 866 | 18 |
|  | M25L | TGGGTTACGATTTTCGCCGC |  |  |
| CTX-M Group 9 (including Toho-2) | M9U | ATGGTGACAAAGAGAGTGCA | 864 | 18 |
|  | M9L | CCCTTCGGCGATGATTCTC |  |  |

| Carbapenemase genes – multiplex PCR (36 cycles of 94°C 30 sec, 52°C 40 sec, and 72°C 50 sec) | | | | |
| --- | --- | --- | --- | --- |
| Target gene | **Primer name** | **Sequence (5′–3′)** | **Amplicon size (bp)** | **Reference** |
| *bla*_IMP_ | IMP-F | GGAATAGAGTGGCTTAAYTCTC | 232 | 17 |
|  | IMP-R | GGTTTAAYAAAACAACCACC |  |  |
| *bla*_NDM_ | NDM-F | GGTTTGGCGATCTGGTTTTC | 621 | 17 |
|  | NDM-R | CGGAATGGCTCATCACGATC |  |  |
| *bla*_OXA-48_ | OXA-F | GCGTGGTTAAGGATGAACAC | 438 | 17 |
|  | OXA-R | CATCAAGTTCAACCCAACCG |  |  |
| *bla*_KPC_ | KPC-F | CGTCTAGTTCTGCTGTCTTG | 798 232 | 17 |
|  | KPC-R | CTTGTCATCCTTGTTAGGCG |  |  |

| *E. coli* ST131 clade / subclade multiplex PCR (30 cycles of 98°C 10 sec, 57°C 20sec, and 72°C 40 sec) | | | | |
| --- | --- | --- | --- | --- |
| Target gene | **Primer name** | **Sequence (5′–3′)** | **Amplicon size (bp)** | **Reference** |
| Clade A-specific (region 4) | CladeAspe4-YF5 | TGACGGGACGTGAGCAAATTA | 707 | 20 |
|  | CladeAspe4-YR5 | AGTCAGACCTAGCCACCCTT |  |  |
| ST131-specific (region 19) | ST131_R19-YF1 | AGCAACGATATTTGCCCATT | 580 | 20 |
|  | ST131_R19-YR1 | GGCGATAACAGTACGCCATT |  |  |
| Clade B-specific (prfC SNP) | prfC-1615spe0-YF1 | CAACGTTGAAGCAGTGTATGAG | 442 | 20 |
|  | prfC-d2034-YR1 | TGACAATCGACGGCTTTAGA |  |  |
| Clade C1 | C1-578spe-YF1 | GGCCCCACAAATTGCTT | 337 | 20 |
|  | C1-898-YR1 | CGCACCTCCGATACCAAA |  |  |
| Subclade C1-M27 (M27PP1) | M27PP1C-YF1 | TGAATCAAAGGTCCGAGCTG | 232 | 20 |
|  | M27PP1C-YR1 | TATGGCTGGCAGATGCTTTA |  |  |
| Clade C2 (nrdI SNP) | nrdI-534spe2-YF1 | ACGGATTCAGGTAGACGATT | 164 | 20 |
|  | nrdI-678R | CCTCACCAAAGTTGCGATTAC |  |  |
| Clade C (mgtA SNP) | C-SNP1-700spe-YF1 | CGCTGGCCAGTTATCTGAAAT | 103 | 20 |
|  | C-SNP1-762spe-YR2 | CCTTTCACCAACTGGGTTACT |  |  |
| Subclade C1-M27 (aer SNP) – confirmatory assay | M27aer-spe-YF1 | GCCGATGGGCTTTCCT | 140 | 20 |
|  | M27aer-YR2 | GTCACCGCGTCTTCCAGT |  |  |

| *E. coli* ST1193 PCR (40 cycles of 98°C 10sec, 62°C 5 sec, 68°C 1 sec) | | | | |
| --- | --- | --- | --- | --- |
| Target gene | **Primer name** | **Sequence (5′–3′)** | **Amplicon size (bp)** | **Reference** |
| icd-200 (ST1193-specific) | 1193icdF.21 | ATTCCTGCGTGAAGAGATGGA | 600 | 19 |
|  | icdgpVII.r | CAATTAAATCAGCCGCTTCG |  |  |

| ST131 and ST1193 O: H typing PCR   - O25b-PCR: 30 cycles of 95°C 30 sec, 60°C sec 30 sec, 72°C 30 sec - Others: 25 cycles of 94°C 30sec, 58°C 30 sec, 72°C 1 min | | | | |
| --- | --- | --- | --- | --- |
| Target gene | **Primer name** | **Sequence (5′–3′)** | **Amplicon size (bp)** | **Reference** |
| O25b allele-specific | rfb.1bis | ATACCGACGACGCCGATCTG | 300 | 23 |
|  | rfbO25b.r | TGCTATTCATTATGCGCAGC |  |  |
| O16 | Og16-PCR_F | GGTTTCAATCTCACAGCAACTCAG | 302 | 22 |
|  | Og16-PCR_R | GTTAGAGGGATAATAGCCAAGCGG |  |  |
| O75 | Og75-PCR_F | GAGATATACATGGGGAGGTAGGCT | 511 | 22 |
|  | Og75-PCR_R | ACCCGATAATCATATTCTTCCCAAC |  |  |
| H4 | Hg4-PCR-F | GATTTCAGCGCGGCGAAACT | 150 | 21 |
|  | Hg4-PCR-R | GGTTGCAGAATCAACGACCG |  |  |
| H5 | Hg5-PCR-F | CGCGTCGATTAATCATACAG | 225 | 21 |
|  | Hg5-PCR-R | GTTGCTTTTGCCGCAGTATT |  |  |

**^1)^** Y=T or C; R=A or G; S=G or C; D=A or G or T.

Supplementary Table S3. Antimicrobial-resistant rates of 1,466 *E. coli* including target isolates of CTX- and CIP- resistant subpopulations.

| Antibiotics | Species | | | | | | | | Total  (n = 1,466) |
| --- | --- | --- | --- | --- | --- | --- | --- | --- | --- |
|  | Camel  (n = 22)^1)^ | Goat  (n = 185) | Sheep  (n = 163) | Cattle  (n = 224) | Horse  (n = 115) | Pig  (n = 172) | Chicken  (n = 181) | Dog  (n = 404) |  |
| GEM | 0 | 0 | 0 | 11  (4.9)^2)^ | 19  (16.5) | 10  (5.8) | 32  (17.7) | 84  (20.8) | 156  (10.6) |
| TET | 0 | 0 | 4  (2.5) | 24  (10.7) | 48  (41.7) | 85  (49.4) | 97  (53.6) | 183  (45.3) | 441  (30.1) |
| CHL | 0 | 0 | 0 | 6  (2.7) | 54  (47.0) | 29  (16.9) | 86  (47.5) | 51  (12.6) | 226  (15.4) |
| AMP | 0 | 0 | 3  (1.8) | 18  (8.0) | 62  (53.9) | 64  (37.2) | 168  (92.8) | 248  (61.4) | 563  (38.4) |
| CFZ | 0 | 0 | 0 | 10  (4.5) | 55  (47.8) | 36  (20.9) | 103  (56.9) | 171  (42.3) | 375  (25.6) |
| CMZ | 0 | 0 | 0 | 0 | 0 | 0 | 0 | 12  (3.0) | 12  (0.8) |
| CFX | 0 | 0 | 0 | 0 | 0 | 0 | 0 | 26  (6.4) | 26  (1.8) |
| CTX | 0 | 0 | 0 | 10  (4.5) | 53  (46.1) | 34  (19.8) | 103  (56.9) | 169  (41.8) | 369  (25.2) |
| MEM | 0 | 0 | 0 | 0 | 0 | 0 | 0 | 15  (3.7) | 15  (1.0) |
| NAL | 0 | 0 | 0 | 13  (5.8) | 33  (28.7) | 80  (46.5) | 138  (76.2) | 157  (38.9) | 421  (28.7) |
| CIP | 0 | 0 | 0 | 8  (3.6) | 29  (25.2) | 49  (28.4) | 101  (55.8) | 110  (27.2) | 311  (21.2) |
| STX | 0 | 0 | 0 | 12  (5.4) | 56  (48.7) | 46  (26.7) | 78  (43.1) | 174  (43.1) | 366  (25.0) |

^1)^ Number of *E. coli* isolated using antibiotic-free (unbiased isolation) and CTX- or CIP-containing ECC (target isolation of CTX- and CIP-resistant subpopulations) (Supplementary Table S1).

^2)^ Numbers in parentheses indicate percentages of AMR *E. coli* isolates to the *E. coli* isolated using antibiotics-free and antibiotics-containing ECC.

Supplementary Table S4. AMR *E. coli* carrying rates in each animal species.

| Antibiotics | Species | | | | | | | | Total^1)^  (n = 403) |
| --- | --- | --- | --- | --- | --- | --- | --- | --- | --- |
|  | Camel  (n = 11) | Goat  (n = 65) | Sheep  (n = 55) | Cattle  (n = 80) | Horse  (n = 26) | Pig  (n = 36) | Chicken  (n = 32) | Dog  (n = 98) |  |
| GEM | 0 | 0 | 0 | 5  (6.3) | 3  (11.5) | 6  (16.7) | 9  (28.1) | 23  (23.5) | 46  (11.4) |
| TET | 0 | 0 | 2  (3.6) | 15  (18.8) | 11  (42.3) | 23  (63.9) | 25  (78.1) | 49  (50.0) | 125  (31.0) |
| CHL | 0 | 0 | 0 | 4  (5.0) | 10  (38.5) | 11  (30.6) | 14  (43.8) | 17  (17.3) | 56  (13.9) |
| AMP | 0 | 0 | 1  (1.8) | 10  (12.5) | 13  (50.0) | 18  (50.0) | 31  (96.9) | 57  (58.2) | 130  (32.3) |
| CFZ | 0 | 0 | 0 | 4  (5.0) | 11  (42.3) | 13  (36.1) | 24  (75.0) | 41  (41.8) | 93  (23.1) |
| CMZ | 0 | 0 | 0 | 0 | 0 | 0 | 0 | 4  (4.1) | 4  (1.0) |
| CFX | 0 | 0 | 0 | 0 | 0 | 0 | 0 | 7  (7.1) | 7  (1.7) |
| CTX | 0 | 0 | 0 | 4  (5.0) | 10  (38.5) | 12  (33.3) | 25  (78.1) | 42  (42.9) | 93  (23.1) |
| MEM | 0 | 0 | 0 | 0 | 0 | 0 | 0 | 2  (2.0) | 2  (0.5) |
| NAL | 0 | 0 | 0 | 8  (10.0) | 5  (19.2) | 18  (50.0) | 22  (68.8) | 41  (41.8) | 94  (23.3) |
| CIP | 0 | 0 | 0 | 5  (5.0) | 4  (15.4) | 15  (41.7) | 15  (46.9) | 26  (26.5) | 65  (16.1) |
| STX | 0 | 0 | 0 | 6  (7.5) | 10  (38.5) | 16  (44.4) | 23  (71.9) | 48  (49.0) | 103  (25.6) |

^1)^ Animal from which at least one *E. coli* was isolated using antibiotic-free (unbiased isolation) and/or antibiotic-containing ECC plates (target isolation of CTX- and CIP-resistant subpopulations) were used for calculation.

^2)^ Even if more than two different types of AMR *E. coli* were isolated from one animal, e.g., different multi-drug resistance patterns, the animal was counted as one AMR *E. coli*-positive animal in each antibiotic.

Supplementary Table S5. Antimicrobial resistance profiles of MDR *E. coli.*^1)^

| No | MDR | Resistant profiles^2)^ | | | | | | | | Cattle | Horse | Pig | Chicken | Dog |
| --- | --- | --- | --- | --- | --- | --- | --- | --- | --- | --- | --- | --- | --- | --- |
| 1 | 3 | PEN | CEP | SUL |  |  |  |  |  | 0 | 0 | 3 | 3 | 5 |
| 2 |  | AG | PEN | CEP |  |  |  |  |  | 0 | 0 | 0 | 0 | 2 |
| 3 |  | AG | TET | PEN |  |  |  |  |  | 0 | 0 | 3 | 0 | 9 |
| 4 |  | TET | PEN | CEP |  |  |  |  |  | 1 | 0 | 5 | 4 | 17 |
| 5 |  | TET | PEN | SUL |  |  |  |  |  | 0 | 0 | 2 | 5 | 10 |
| 6 |  | TET | CMP | SUL |  |  |  |  |  | 0 | 0 | 1 | 0 | 0 |
| 7 |  | TET | FQ | PEN |  |  |  |  |  | 0 | 0 | 4 | 1 | 0 |
| 8 |  | TET | FQ | SUL |  |  |  |  |  | 0 | 0 | 0 | 0 | 1 |
| 9 |  | CMP | PEN | SUL |  |  |  |  |  | 0 | 0 | 2 | 0 | 0 |
| 10 |  | FQ | PEN | CEP |  |  |  |  |  | 0 | 0 | 2 | 6 | 2 |
| 11 |  | FQ | PEN | SUL |  |  |  |  |  | 0 | 0 | 0 | 1 | 10 |
| 12 | 4 | AG | TET | PEN | SUL |  |  |  |  | 0 | 0 | 1 | 0 | 5 |
| 13 |  | AG | PEN | CEP | SUL |  |  |  |  | 0 | 0 | 0 | 0 | 3 |
| 14 |  | AG | FQ | PEN | CEP |  |  |  |  | 0 | 0 | 0 | 0 | 3 |
| 15 |  | AG | FQ | PEN | SUL |  |  |  |  | 0 | 0 | 0 | 0 | 1 |
| 16 |  | AG | TET | CMP | PEN |  |  |  |  | 0 | 0 | 0 | 0 | 1 |
| 17 |  | AG | TET | FQ | PEN |  |  |  |  | 0 | 0 | 3 | 0 | 0 |
| 18 |  | TET | PEN | CEP | SUL |  |  |  |  | 0 | 3 | 1 | 5 | 25 |
| 19 |  | TET | CMP | PEN | CEP |  |  |  |  | 0 | 0 | 0 | 0 | 6 |
| 20 |  | TET | CMP | PEN | SUL |  |  |  |  | 0 | 3 | 0 | 3 | 7 |
| 21 |  | TET | CMP | FQ | PEN |  |  |  |  | 0 | 0 | 0 | 22 | 0 |
| 22 |  | TET | CMP | FQ | SUL |  |  |  |  | 0 | 0 | 10 | 0 | 0 |
| 23 |  | TET | FQ | PEN | CEP |  |  |  |  | 0 | 0 | 6 | 0 | 5 |
| 24 |  | TET | FQ | PEN | SUL |  |  |  |  | 0 | 0 | 0 | 2 | 6 |
| 25 |  | CMP | PEN | CEP | SUL |  |  |  |  | 0 | 3 | 0 | 0 | 0 |
| 26 |  | CMP | FQ | PEN | CEP |  |  |  |  | 0 | 0 | 0 | 1 | 0 |
| 27 |  | CMP | FQ | PEN | SUL |  |  |  |  | 0 | 0 | 6 | 0 | 0 |
| 28 |  | FQ | PEN | CEP | SUL |  |  |  |  | 0 | 0 | 1 | 0 | 3 |
| 29 | 5 | AG | TET | PEN | CEP | SUL |  |  |  | 7 | 0 | 1 | 0 | 10 |
| 30 |  | AG | TET | CMP | PEN | SUL |  |  |  | 1 | 0 | 0 | 0 | 0 |
| 31 |  | AG | CMP | PEN | CEP | SUL |  |  |  | 0 | 3 | 0 | 0 | 0 |
| 32 |  | AG | CMP | FQ | PEN | CEP |  |  |  | 0 | 0 | 0 | 10 | 0 |
| 33 |  | AG | FQ | PEN | CEP | SUL |  |  |  | 0 | 0 | 0 | 5 | 9 |
| 34 |  | AG | TET | FQ | PEN | CEP |  |  |  | 0 | 0 | 1 | 0 | 0 |
| 35 |  | AG | TET | FQ | PEN | SUL |  |  |  | 0 | 0 | 0 | 0 | 5 |
| 36 |  | CMP | FQ | PEN | CEP | SUL |  |  |  | 0 | 8 | 3 | 2 | 0 |
| 37 |  | TET | CMP | PEN | CEP | SUL |  |  |  | 0 | 19 | 3 | 4 | 5 |
| 38 |  | TET | CMP | FQ | PEN | SUL |  |  |  | 0 | 0 | 0 | 17 | 2 |
| 39 |  | TET | FQ | PEN | CEP | SUL |  |  |  | 0 | 0 | 3 | 3 | 22 |
| 40 |  | TET | CMP | FQ | CEP | SUL |  |  |  | 0 | 0 | 1 | 0 | 0 |
| 41 | 6 | AG | TET | CMP | PEN | CEP | SUL |  |  | 0 | 0 | 0 | 0 | 3 |
| 42 |  | AG | CMP | FQ | PEN | CEP | SUL |  |  | 0 | 0 | 0 | 3 | 0 |
| 43 |  | AG | TET | CMP | FQ | PEN | CEP |  |  | 0 | 1 | 0 | 1 | 0 |
| 44 |  | AG | TET | CMP | FQ | PEN | SUL |  |  | 3 | 0 | 0 | 0 | 1 |
| 45 |  | AG | TET | FQ | PEN | CEP | SUL |  |  | 0 | 0 | 0 | 1 | 9 |
| 46 |  | TET | CMP | FQ | PEN | CEP | SUL |  |  | 0 | 1 | 3 | 11 | 3 |
| 47 | 7 | AG | TET | CMP | FQ | PEN | CEP | SUL |  | 0 | 15 | 0 | 12 | 6 |
| 48 | 8 | AG | TET | CMP | FQ | PEN | CEP | CP | SUL | 0 | 0 | 0 | 0 | 15 |
| Total | | | | | | | | | | 12 | 56 | 65 | 122 | 211 |

^1)^ Total *E. coli* isolates using antibiotic-free (unbiased isolation) and antibiotic-containing CHROMAgar ECC plates (target isolation of CTX- and CIP-resistant subpopulations) were used for assignment (n = 1,466). Isolates which show resistance to 3 or more different antibiotic classes were considered as MDR *E. coli*.

^2)^ AG, Aminoglycosides; TET, Tetracyclines; CMP, Amphenicols; FQ, Fluoroquinolones; PEN, Penicillins; CEP, Cephalosporines; CP, Carbapenems; SUL: Sulfonamides.

Supplementary Table S6. Identification of *bla*_CTX-M_ gene subtypes among CEP-resistant *E. coli* isolates

| CTX-M family | CTX-M subtype | Cattle  (n = 5) ^1)^ | Horse  (n = 19) | Pig  (n = 10) | Chicken  (n = 30) | Dog  (n = 59) | Total  (n = 123) |
| --- | --- | --- | --- | --- | --- | --- | --- |
| *bla*_CTX-M-1_-like  (n = 80) | *bla*_CTX-M-1_ | 0^2)^ | 5 | 0 | 0 | 0 | 5 |
|  | *bla*_CTX-M-3_ | 0 | 0 | 0 | 0 | 4 | 4 |
|  | *bla*_CTX-M-15_ | 1 | 1 | 2 | 0 | 16 | 20 |
|  | *bla*_CTX-M-55_ | 4 | 0 | 2 | 24 | 15 | 45 |
|  | *bla*_CTX-M-64_ | 0 | 0 | 0 | 0 | 6 | 6 |
| *bla*_CTX-M-9_-like  (n = 43) | *bla*_CTX-M-14_ | 0 | 0 | 2 | 5 | 2 | 9 |
|  | *bla*_CTX-M-27_ | 0 | 0 | 2 | 0 | 14 | 16 |
|  | *bla*_CTX-M-65_ | 0 | 13 | 2 | 1 | 2 | 18 |

^1)^ Number of *bla*_CTX-M_ family gene-positive *E. coli* used for each species.

^2)^ Number of *bla*_CTX-M_ subtype-positive *E. coli* isolates.
