# Supplementary material for: Prevalence of antimicrobial-resistant Escherichia coli as an indicator bacterium in livestock and companion animals in Mongolia
Source: One Health. 2026 Jun 27;23:101502. doi: 10.1016/j.onehlt.2026.101502 (PMC13330623; doi:10.1016/j.onehlt.2026.101502)
Supplement: Supplementary file 2 — Supplementary material 2 [file mmc2.pdf]

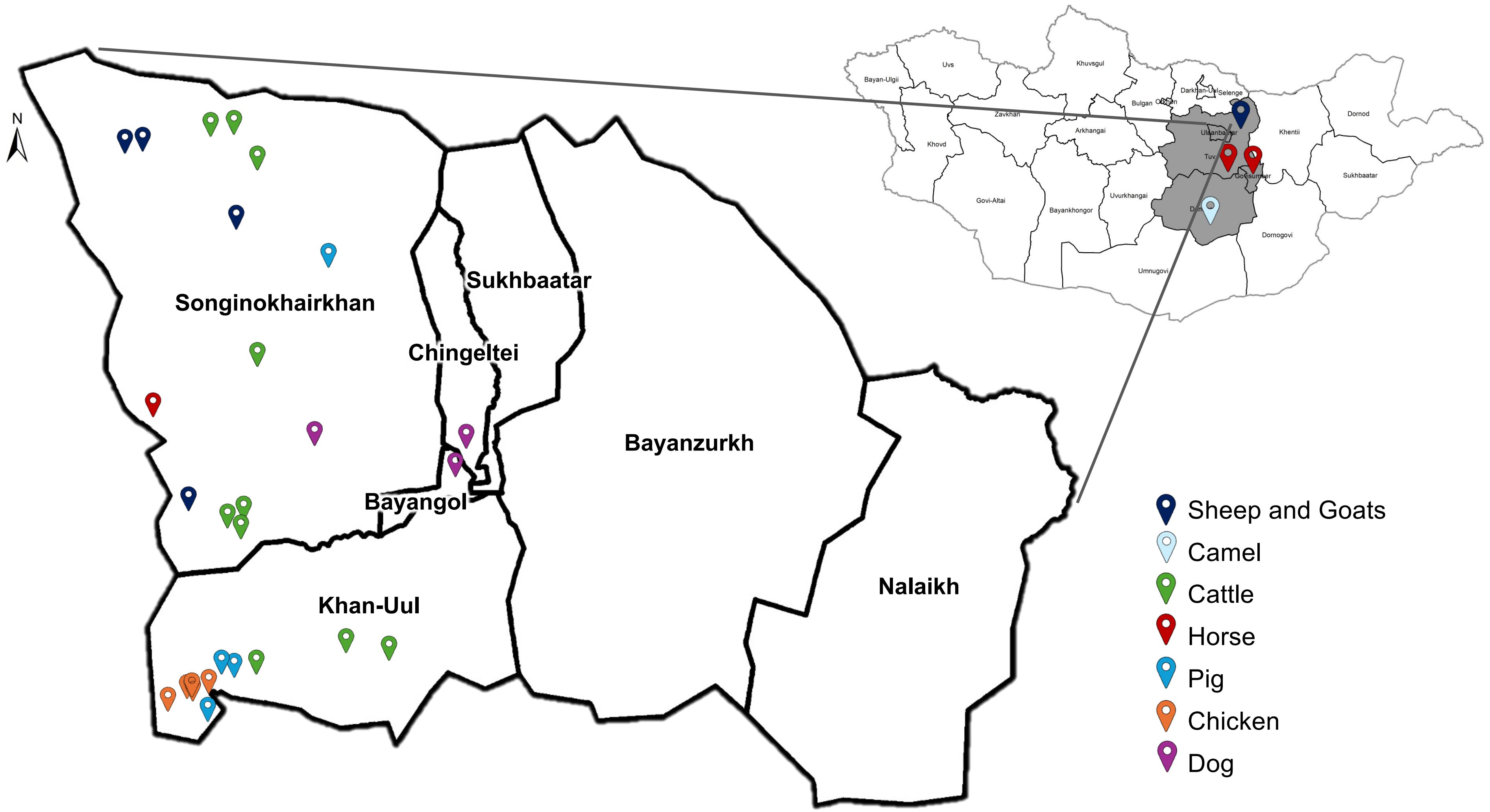

Supplementary Figure S1. Locations of sampling sites. Grey colored areas indicate the sampling areas. Colored markers represent animal species.

Supplementary Table S1. Isolation of *E. coli* from various animal species in Mongolia.

Species	Farm No.	Animal No. used for isolation	Animal No. *E. coli* isolated	Isolate number (number of animal)			
				ECC[1)]	ECC-CTX[2)]	ECC-CIP[3)]	Total
Camel	1	19	11	22 (11)	0 (0)	0 (0)	22
Goat	6	69	65	185 (65)	0 (0)	0 (0)	185
Sheep	6	57	55	163 (55)	0 (0)	0 (0)	163
Cattle	10	100	80	211 (80)	10 (4)	3 (1)	224
Horse	3[4)]	30	26	73 (25)	30 (10)	12 (4)	115
Pig	4	40	36	106 (36)	24 (9)	42 (15)	172
Chicken	5	50	32	90 (31)	54 (19)	37 (13)	181
Dog	4[5)]	154	98	260 (95)	86 (35)	58 (20)	404
Total	39	519	403	1,110 (398)	204 (77)	152 (53)	1,466

[1)] Isolates using CHROMAgar ECC agar plate without antibiotics that were used for calculating unbiased AMR rates.

[2)] Isolates using CHROMAgar ECC agar plate supplemented with 1μg/mL cefotaxime (CTX) that were used as targeted isolates of CTX-resistant subpopulations.

[3)] Isolates using CHROMAgar ECC agar plate supplemented with 1μg/mL ciprofloxacin (CIP) that were used as targeted isolates of CIP-resistant subpopulations.

[4)] Number of clinics and farms where rectal swabs were collected

[5)] Total number of clinics and a dog shelter where rectal swabs were collected

Supplementary Table 2. Primer sequences and expected amplicon sizes for each PCR primer set used in this study.

Multiplex/simplex PCR for major β-lactamase genes (30 cycles of 94°C 40 sec, 60°C 40 sec, 72°C for 1 min):<br>- 55°C for amplification of *bla*VIM, *bla*IMP and *bla*KPC genes<br>- 57°C for amplification of *bla*GES and *bla*OXA-48 genes				
**Target gene**	**Primer name**	**Sequence (5′–3′)**	**Amplicon size (bp)**	**Reference**
**TEM variants**	MultiTSO-T_for<br>MultiTSO-T_rev	CATTTCCTGTCGGCCTTATTC<br>CGTTCATCCATAGTTGCCTGA	800	Dallenne et al. 2010
**SHV variants**	MultiTSO-S_for<br>MultiTSO-S_rev	AGCCGCTTGAGCAAATTAAAC<br>ATCCCGCAGATAAATCACCAC	713	Dallenne et al. 2010
**OXA-1-like**	MultiTSO-O_for<br>MultiTSO-O_rev	GGCACCAGATTCAACTTTCAAG<br>GACCCCAAGTTTCCTGTAAGTG	640	Dallenne et al. 2010
**CTX-M Group 1**	MultiCTXM Gp1_for<br>MultiCTXM Gp1-2_rev	TTAGGAARTGTGCCGCTGYA[1)]<br>CGATATCGTTGGTGGTTRCCAT	688	Dallenne et al. 2010
**CTX-M Group 2**	MultiCTXM Gp2_for<br>MultiCTXM Gp1-2_rev	CGTTAACGGCACGATGAC<br>CGATATCGTTGGTGGTTRCCAT	404	Dallenne et al. 2010
**CTX-M Group 9**	MultiCTXM Gp9_for<br>MultiCTXM Gp9_rev	TCAAGCCTGCCGATCTGGT<br>TGATTCTCGCCGCTGAAG	561	Dallenne et al. 2010
**CTX-M Group 8/25**	CTX-M Gp8/25-F<br>CTX-M Gp8/25-R	AACRCRCAGACGCTCTAC<br>TCGAGCCGGAASGTGTYAT	326	Dallenne et al. 2010
**DHA-1 and DHA-2**	MultiCaseDHA_for<br>MultiCaseDHA_rev	TGATGGCACAGCAGGATATTC<br>GCTTTGACTCTTTCGGTATTCG	997	16
**CMY-2 group (CIT)**	MultiCaseCIT_for<br>MultiCaseCIT_rev	CGAAGAGGCAATGACCAGAC<br>ACGGACAGGGTTAGGATAGY	538	16
**EBC (AmpC)**	MultiCaseEBC_for<br>MultiCaseEBC_rev	CGGTAAAGCCGATGTTGCG<br>AGCCTAACCCCTGATACA	683	16
**GES-1 to GES-9, GES-11**	MultiGES_for<br>MultiGES_rev	AGTCGGCTAGACCGGAAAG<br>TTTGTCCGTGCTCAGGAT	399	16
**PER-1, PER-3**	MultiPER_for<br>MultiPER_rev	GCTCCGATAATGAAAGCGT<br>TTCGGCTTGACTCGGCTGA	520	16
**VEB-1 to VEB-6**	MultiVEB_for<br>MultiVEB_rev	CATTTCCCGATGCAAAGCGT<br>CGAAGTTTCTTTGGACTCTG	648	16
**GES-1 to GES-9, GES-11**	MultiGES_for<br>MultiGES_rev	AGTCGGCTAGACCGGAAAG<br>TTTGTCCGTGCTCAGGAT	399	16
**OXA-48-like**	MultiOXA-48_for<br>MultiOXA-48_rev	GCTTGATCGCCCTCGATT<br>GATTTGCTCCGTGGCCGAAA	281	16
**IMP variants**	MultiIMP_for<br>MultiIMP_rev	TTGACACTCCATTTACDG<br>GATYGAGAATTAAGCCACYCT	139	16
**VIM variants (including VIM-1, VIM-2)**	MultiVIM_for<br>MultiVIM_rev	GATGGTGTTTGGTCGCATA<br>CGAATGCGCAGCACCAG	390	16
**KPC-1 to KPC-5**	MultiKPC_for<br>MultiKPC_rev	CATTCAAGGGCTTTCTTGCTGC<br>ACGACGGCATAGTCATTTGC	538	16

*bla*CTX-M PCR for group 1/2/9 typing - DNA sequencing (30 cycles of 95°C 30 sec, 60°C 30sec, and 72°C 1min)				
**Target gene**	**Primer name**	**Sequence (5′–3′)**	**Amplicon size (bp)**	**Reference**
**CTX-M Group 1**	M13U<br>M13L	GGTTAAAAAATCACTGCGTC<br>TTGGTGACGATTTTAGCCGC	864	18
**CTX-M Group 2**	M25U	ATGATGACTCAGAGCATTCG	866	18

	M25L	TGGGTTACGATTTTCGCCGC		
**CTX-M Group 9 (including Toho-2)**	M9U<br>M9L	ATGGTGACAAAGAGAGTGCA<br>CCCTTCGGCGATGATTCTC	864	18

Carbapenemase genes – multiplex PCR (36 cycles of 94°C 30 sec, 52°C 40 sec, and 72°C 50 sec)

Target gene	Primer name	Sequence (5′–3′)	Amplicon size (bp)	Reference
***bla*IMP**	IMP-F<br>IMP-R	GGAATAGAGTGGCTTAAYTCTC<br>GGTTTAAYAAAACAACCACC	232	17
***bla*NDM**	NDM-F<br>NDM-R	GGTTTGGCGATCTGGTTTTC<br>CGGAATGGCTCATCACGATC	621	17
***bla*OXA-48**	OXA-F<br>OXA-R	GCGTGGTTAAGGATGAACAC<br>CATCAAGTTCAACCCAACCG	438	17
***bla*KPC**	KPC-F<br>KPC-R	CGTCTAGTTCTGCTGTCTTG<br>CTTGTCATCCTTGTTAGGCG	798<br>232	17

***E. coli* ST131 clade / subclade multiplex PCR (30 cycles of 98°C 10 sec, 57°C 20sec, and 72°C 40 sec)**

Target gene	Primer name	Sequence (5′–3′)	Amplicon size (bp)	Reference
**Clade A-specific (region 4)**	CladeAspe4-YF5<br>CladeAspe4-YR5	TGACGGGACGTGAGCAAATTA<br>AGTCAGACCTAGCCACCCTT	707	20
**ST131-specific (region 19)**	ST131_R19-YF1<br>ST131_R19-YR1	AGCAACGATATTTGCCCATT<br>GGCGATAACAGTACGCCATT	580	20
**Clade B-specific (prfC SNP)**	prfC-1615spe0-YF1<br>prfC-d2034-YR1	CAACGTTGAAGCAGTGTATGAG<br>TGACAATCGACGGCTTTAGA	442	20
**Clade C1**	C1-578spe-YF1<br>C1-898-YR1	GGCCCCACAAATTGCTT<br>CGCACCTCCGATACCAAA	337	20
**Subclade C1-M27 (M27PP1)**	M27PP1C-YF1<br>M27PP1C-YR1	TGAATCAAAGGTCCGAGCTG<br>TATGGCTGGCAGATGCTTTA	232	20
**Clade C2 (nrdI SNP)**	nrdI-534spe2-YF1<br>nrdI-678R	ACGGATTCAGGTAGACGATT<br>CCTCACCAAAGTTGCGATTAC	164	20
**Clade C (mgtA SNP)**	C-SNP1-700spe-YF1<br>C-SNP1-762spe-YR2	CGCTGGCCAGTTATCTGAAAT<br>CCTTTCACCAACTGGGTTACT	103	20
**Subclade C1-M27 (aer SNP) – confirmatory assay**	M27aer-spe-YF1<br>M27aer-YR2	GCCGATGGGCTTTCCT<br>GTCACCGCGTCTTCCAGT	140	20

***E. coli* ST1193 PCR (40 cycles of 98°C 10sec, 62°C 5 sec, 68°C 1 sec)**

Target gene	Primer name	Sequence (5′–3′)	Amplicon size (bp)	Reference
**icd-200 (ST1193-specific)**	1193icdF.21<br>icdgpVII.r	ATTCCTGCGTGAAGAGATGGA<br>CAATTAAATCAGCCGCTTCG	600	19

ST131 and ST1193 O: H typing PCR
- O25b-PCR: 30 cycles of 95°C 30 sec, 60°C sec 30 sec, 72°C 30 sec
- Others: 25 cycles of 94°C 30sec, 58°C 30 sec, 72°C 1 min

Target gene	Primer name	Sequence (5′–3′)	Amplicon size (bp)	Reference
**O25b allele-specific**	rfb.1bis<br>rfbO25b.r	ATACCGACGACGCCGATCTG<br>TGCTATTCATTATGCGCAGC	300	23
**O16**	Og16-PCR_F<br>Og16-PCR_R	GGTTTCAATCTCACAGCAACTCAG<br>GTTAGAGGGATAATAGCCAAGCGG	302	22
**O75**	Og75-PCR_F	GAGATATACATGGGGAGGTAGGCT	511	22

	Og75-PCR_R	ACCCGATAATCATATTCTTCCCAAC		
**H4**	Hg4-PCR-F	GATTTCAGCGCGGCGAAACT	150	21
	Hg4-PCR-R	GGTTGCAGAATCAACGACCG		
**H5**	Hg5-PCR-F	CGCGTCGATTAATCATACAG	225	21
	Hg5-PCR-R	GTTGCTTTTGCCGCAGTATT		

[1)] Y=T or C; R=A or G; S=G or C; D=A or G or T.

Supplementary Table S3. Antimicrobial-resistant rates of 1,466 *E. coli* including target isolates of CTX- and CIP- resistant subpopulations.

Antibiotics	Species								Total (n = 1,466)
	Camel (n = 22)[1)]	Goat (n = 185)	Sheep (n = 163)	Cattle (n = 224)	Horse (n = 115)	Pig (n = 172)	Chicken (n = 181)	Dog (n = 404)	
GEM	0	0	0	11 (4.9)[2)]	19 (16.5)	10 (5.8)	32 (17.7)	84 (20.8)	156 (10.6)
TET	0	0	4 (2.5)	24 (10.7)	48 (41.7)	85 (49.4)	97 (53.6)	183 (45.3)	441 (30.1)
CHL	0	0	0	6 (2.7)	54 (47.0)	29 (16.9)	86 (47.5)	51 (12.6)	226 (15.4)
AMP	0	0	3 (1.8)	18 (8.0)	62 (53.9)	64 (37.2)	168 (92.8)	248 (61.4)	563 (38.4)
CFZ	0	0	0	10 (4.5)	55 (47.8)	36 (20.9)	103 (56.9)	171 (42.3)	375 (25.6)
CMZ	0	0	0	0	0	0	0	12 (3.0)	12 (0.8)
CFX	0	0	0	0	0	0	0	26 (6.4)	26 (1.8)
CTX	0	0	0	10 (4.5)	53 (46.1)	34 (19.8)	103 (56.9)	169 (41.8)	369 (25.2)
MEM	0	0	0	0	0	0	0	15 (3.7)	15 (1.0)
NAL	0	0	0	13 (5.8)	33 (28.7)	80 (46.5)	138 (76.2)	157 (38.9)	421 (28.7)
CIP	0	0	0	8 (3.6)	29 (25.2)	49 (28.4)	101 (55.8)	110 (27.2)	311 (21.2)
STX	0	0	0	12 (5.4)	56 (48.7)	46 (26.7)	78 (43.1)	174 (43.1)	366 (25.0)

1) Number of *E. coli* isolated using antibiotic-free (unbiased isolation) and CTX- or CIP-containing ECC (target isolation of CTX- and CIP-resistant subpopulations) (Supplementary Table S1).

2) Numbers in parentheses indicate percentages of AMR *E. coli* isolates to the *E. coli* isolated using antibiotics-free and antibiotics-containing ECC.

Supplementary Table S4. AMR *E. coli* carrying rates in each animal species.

Antibiotics	Species								Total[1)] (n = 403)
	Camel (n = 11)	Goat (n = 65)	Sheep (n = 55)	Cattle (n = 80)	Horse (n = 26)	Pig (n = 36)	Chicken (n = 32)	Dog (n = 98)	
GEM	0	0	0	5 (6.3)	3 (11.5)	6 (16.7)	9 (28.1)	23 (23.5)	46 (11.4)
TET	0	0	2 (3.6)	15 (18.8)	11 (42.3)	23 (63.9)	25 (78.1)	49 (50.0)	125 (31.0)
CHL	0	0	0	4 (5.0)	10 (38.5)	11 (30.6)	14 (43.8)	17 (17.3)	56 (13.9)
AMP	0	0	1 (1.8)	10 (12.5)	13 (50.0)	18 (50.0)	31 (96.9)	57 (58.2)	130 (32.3)
CFZ	0	0	0	4 (5.0)	11 (42.3)	13 (36.1)	24 (75.0)	41 (41.8)	93 (23.1)
CMZ	0	0	0	0	0	0	0	4 (4.1)	4 (1.0)
CFX	0	0	0	0	0	0	0	7 (7.1)	7 (1.7)
CTX	0	0	0	4 (5.0)	10 (38.5)	12 (33.3)	25 (78.1)	42 (42.9)	93 (23.1)
MEM	0	0	0	0	0	0	0	2 (2.0)	2 (0.5)
NAL	0	0	0	8 (10.0)	5 (19.2)	18 (50.0)	22 (68.8)	41 (41.8)	94 (23.3)
CIP	0	0	0	5 (5.0)	4 (15.4)	15 (41.7)	15 (46.9)	26 (26.5)	65 (16.1)
STX	0	0	0	6 (7.5)	10 (38.5)	16 (44.4)	23 (71.9)	48 (49.0)	103 (25.6)

[1)] Animal from which at least one *E. coli* was isolated using antibiotic-free (unbiased isolation) and/or antibiotic-containing ECC plates (target isolation of CTX- and CIP-resistant subpopulations) were used for calculation.

[2)] Even if more than two different types of AMR *E. coli* were isolated from one animal, e.g., different multi-drug resistance patterns, the animal was counted as one AMR *E. coli*-positive animal in each antibiotic.

Supplementary Table S5. Antimicrobial resistance profiles of MDR *E. coli.*[1)]

No	MDR	Resistant profiles[2)]								Cattle	Horse	Pig	Chicken	Dog
1		PEN	CEP	SUL						0	0	3	3	5
2		AG	PEN	CEP						0	0	0	0	2
3		AG	TET	PEN						0	0	3	0	9
4		TET	PEN	CEP						1	0	5	4	17
5		TET	PEN	SUL						0	0	2	5	10
6	3	TET	CMP	SUL						0	0	1	0	0
7		TET	FQ	PEN						0	0	4	1	0
8		TET	FQ	SUL						0	0	0	0	1
9		CMP	PEN	SUL						0	0	2	0	0
10		FQ	PEN	CEP						0	0	2	6	2
11		FQ	PEN	SUL						0	0	0	1	10
12		AG	TET	PEN	SUL					0	0	1	0	5
13		AG	PEN	CEP	SUL					0	0	0	0	3
14		AG	FQ	PEN	CEP					0	0	0	0	3
15		AG	FQ	PEN	SUL					0	0	0	0	1
16		AG	TET	CMP	PEN					0	0	0	0	1
17		AG	TET	FQ	PEN					0	0	3	0	0
18		TET	PEN	CEP	SUL					0	3	1	5	25
19		TET	CMP	PEN	CEP					0	0	0	0	6
20	4	TET	CMP	PEN	SUL					0	3	0	3	7
21		TET	CMP	FQ	PEN					0	0	0	22	0
22		TET	CMP	FQ	SUL					0	0	10	0	0
23		TET	FQ	PEN	CEP					0	0	6	0	5
24		TET	FQ	PEN	SUL					0	0	0	2	6
25		CMP	PEN	CEP	SUL					0	3	0	0	0
26		CMP	FQ	PEN	CEP					0	0	0	1	0
27		CMP	FQ	PEN	SUL					0	0	6	0	0
28		FQ	PEN	CEP	SUL					0	0	1	0	3
29		AG	TET	PEN	CEP	SUL				7	0	1	0	10
30		AG	TET	CMP	PEN	SUL				1	0	0	0	0
31		AG	CMP	PEN	CEP	SUL				0	3	0	0	0
32		AG	CMP	FQ	PEN	CEP				0	0	0	10	0
33		AG	FQ	PEN	CEP	SUL				0	0	0	5	9
34	5	AG	TET	FQ	PEN	CEP				0	0	1	0	0
35		AG	TET	FQ	PEN	SUL				0	0	0	0	5
36		CMP	FQ	PEN	CEP	SUL				0	8	3	2	0
37		TET	CMP	PEN	CEP	SUL				0	19	3	4	5
38		TET	CMP	FQ	PEN	SUL				0	0	0	17	2
39		TET	FQ	PEN	CEP	SUL				0	0	3	3	22
40		TET	CMP	FQ	CEP	SUL				0	0	1	0	0
41		AG	TET	CMP	PEN	CEP	SUL			0	0	0	0	3
42		AG	CMP	FQ	PEN	CEP	SUL			0	0	0	3	0
43	6	AG	TET	CMP	FQ	PEN	CEP			0	1	0	1	0
44		AG	TET	CMP	FQ	PEN	SUL			3	0	0	0	1
45		AG	TET	FQ	PEN	CEP	SUL			0	0	0	1	9
46		TET	CMP	FQ	PEN	CEP	SUL			0	1	3	11	3
47	7	AG	TET	CMP	FQ	PEN	CEP	SUL		0	15	0	12	6
48	8	AG	TET	CMP	FQ	PEN	CEP	CP	SUL	0	0	0	0	15
		Total								12	56	65	122	211

1) Total *E. coli* isolates using antibiotic-free (unbiased isolation) and antibiotic-containing CHROMAgar ECC plates (target isolation of CTX- and CIP-resistant subpopulations) were used for assignment (n = 1,466). Isolates which show resistance to 3 or more different antibiotic classes were considered as MDR *E. coli*.

2) AG, Aminoglycosides; TET, Tetracyclines; CMP, Amphenicols; FQ, Fluoroquinolones; PEN, Penicillins; CEP, Cephalosporines; CP, Carbapenems; SUL: Sulfonamides.

Supplementary Table S6. Identification of *bla*$_{CTX-M}$ gene subtypes among CEP-resistant *E. coli* isolates

CTX-M family	CTX-M subtype	Cattle (n = 5) [1)]	Horse (n = 19)	Pig (n = 10)	Chicken (n = 30)	Dog (n = 59)	Total (n = 123)
bla$_{CTX-M-1}$-like (n = 80)	*bla*$_{CTX-M-1}$	0[2)]	5	0	0	0	5
	bla$_{CTX-M-3}$	0	0	0	0	4	4
	bla$_{CTX-M-15}$	1	1	2	0	16	20
	bla$_{CTX-M-55}$	4	0	2	24	15	45
	bla$_{CTX-M-64}$	0	0	0	0	6	6
bla$_{CTX-M-9}$-like (n = 43)	*bla*$_{CTX-M-14}$	0	0	2	5	2	9
	bla$_{CTX-M-27}$	0	0	2	0	14	16
	bla$_{CTX-M-65}$	0	13	2	1	2	18

[1)] Number of *bla*$_{CTX-M}$ family gene-positive *E. coli* used for each species.

[2)] Number of *bla*$_{CTX-M}$ subtype-positive *E. coli* isolates.
